# Coaching Parents of Children with Sensory Integration Difficulties: A Scoping Review

**DOI:** 10.1155/2021/6662724

**Published:** 2021-06-17

**Authors:** Susan Allen, Fiona J. Knott, Amanda Branson, Shelly J. Lane

**Affiliations:** ^1^School of Psychology and Clinical Language Science, University of Reading, Henry Pitt Building, Earley Gate, Reading RG6 7BE, UK; ^2^School of Social and Life Sciences, Glyndwr University, Mold Road, Wrexham, Wales, LL13 2AW, UK; ^3^Department of Occupational Therapy, College of Health and Human Sciences, Colorado State University, 1573 Campus Delivery, Fort Collins, CO 80523, USA

## Abstract

**Aim:**

To review current evidence regarding the effectiveness of occupational therapy coaching interventions for parents of children with sensory integration difficulties, delivered to individuals or groups of parents.

**Method:**

A historical scoping review was completed of empirical research records to summarize what is known and how this information can guide future research. The process was guided by PRISMA guidelines. Inclusion criteria were English language and peer-reviewed empirical studies of parent coaching intervention for children with sensory processing or sensory integration difficulties. Five databases were searched. Papers were critically reviewed using McMaster's guidelines.

**Results:**

Four studies met the search criteria. Three studies took a direct coaching approach with individual parents or families. The fourth study took a mixed educational/coaching approach with groups of parents and teachers.

**Conclusion:**

There is some evidence to conclude that occupational therapists can deliver individual parent-focused coaching interventions which impact positively on individual child goals, parental stress, and sense of competence. Group intervention can lead to caregivers' improved perceived and actual knowledge of sensory integration, as well as a sense of self-efficacy in dealing with sensory-related child behaviors. Current evidence is limited. Suggestions for future research are offered.

## 1. Introduction

Sensory integration is the process by which we receive and respond to information through our senses and the way we organize and use this information to participate in everyday activities. In typical development, children gain skills to manage the sensory demands of their bodies and environment to make sense of themselves and their world to interact appropriately [1]. Difficulty in integrating this information enacts a range of processes and responses affecting physiological, cognitive, motor, emotional, and regulatory functions impacting on social relationships and participation in everyday life [2]. The term “sensory processing disorder” is also used to describe difficulty in detecting, regulating, interpreting, and responding to sensory input where difficulties impair daily routines or roles [3]. For the purpose of clarity and in line with the work of Lane et al. [4], the term “sensory integration difficulties” will be used to describe the barriers experienced by individuals with challenges in sensory processing or sensory integration. Occupational therapists' primary concern is how these difficulties impact on the successful participation of children and families in their daily lives [5, 6].

At a policy level in the United Kingdom, the College of Occupational Therapy briefing paper [7] considers intervention for sensory integration difficulties from two perspectives. The first approach is defined as impairment orientated and includes Ayres Sensory Integration, a specialized assessment and direct intervention carried out mainly by occupational therapists with specific postgraduate training using procedural and structural criteria as defined by Parham et al. [8]. Impairment-oriented approaches also include sensory-based interventions, a wide range of sensory stimuli, and sensory experiences using “sensory” equipment in specialized settings. The second approach is defined as performance orientated. This approach emphasizes managing, rather than changing, the sensory needs of the individual through adapting the environment, modifying the task, or developing strategies for the individual to self-manage the task. A variety of interventions have been developed for children and their families with varying effectiveness [9, 10]. This paper explores parent-focused coaching intervention, a relatively underused approach with parents of children with sensory integration difficulties.

Parent engagement in therapy sessions is seen as desirable [11], and at least one child-centered approach explicitly identifies the importance of collaboration and problem-solving with parents [12]. However, impairment-orientated and performance-orientated approaches primarily focus on the child rather than the parent's needs. The impact of sensory integration difficulties on parents has been investigated in a number of papers demonstrating high levels of parent stress as well as challenges to daily occupations. Parents at times use negative coping strategies that can exacerbate the impact of stress for both the parent and the child [13, 14]. Despite research recognizing the impact on parents, there is little evidence about the best way to support parents. Ecological theory [15] reflects the interactive nature of the child with their social and physical environment. Parents experience higher stress and may use negative coping strategies but can nonetheless be positive agents of change for themselves and their children. There is therefore a need to move beyond child-focused work and to explore the value and effectiveness of parent-based interventions. Such interventions include those which increase parental knowledge and understanding to enable parents to gain confidence and competence in developing strategies to better manage their family and children's participation in daily activities.

Two papers have proposed frameworks of intervention for children with sensory integration difficulties and their families. Both frameworks [16, 17] advocate direct interaction with parents using a coaching-based approach, either with parents alone or in addition to intervention with the child or environmental adaptation. It is acknowledged that one approach to intervention cannot meet the whole needs of clients across the variety of settings that we practice in. Multicomponent approaches have the flexibility to allow the needs of children and families to be met in a way which is both cost effective and acceptable to the family. Some parents, for instance, may benefit from support to identify and manage environmental challenges, while for other families, direct intervention with the child may be the most appropriate starting point. We therefore need to consider, develop, and evaluate a variety of intervention approaches in our practice, and coaching is one of these. Coaching has roots in fields outside occupational therapy, e.g., sports and business. It has been simply described as unlocking people's potential to maximize their own performance [18] and is founded upon the relationship between coach and coachee [19]. Reynolds et al. [16] describe coaching as a structured process including emotional support and information exchange. Caregiver interventions are recognized to build on family strengths, occur in natural contexts, and are embedded in daily occupations which support goal acquisition [16]. Caregiver interventions offer the opportunity to empower families in generating strategies compatible with their own routines as well as to be generalized to other situations [17]. Miller-Kuhaneck and Watling [20] systematically reviewed the outcomes of both parent education (didactic teaching) and parent coaching studies for parents of children with autism and sensory integration difficulties and found that although limited there is some evidence to support parent training. They recommend that occupational therapy is well suited to providing parent training. These papers have opened an exploratory door on parent interventions. Notably, they included only one paper on parent coaching. The remaining studies were concerned with teaching parents an activity to apply to the child (e.g., massage or applied sensory stimuli) or addressing sensory integration difficulties as part of a broader intervention for parents of children with autism. Interventions described either did not specifically address the wider population of parents of children with sensory integration difficulties or did not explore coaching as proposed by the recommended frameworks [17].

The overall aim of this study is to review the literature available to support future development of a parent coaching intervention for parents of children with sensory integration difficulties. Specific aims are to synthesize and appraise what we know about parent coaching interventions for parents of children with sensory processing and integration difficulties and to define gaps in the literature to inform future research.

## 2. Methods

To map the literature, identify gaps in our knowledge and understanding of the field, and make recommendations, a scoping review was conducted. PRISMA guidelines for scoping reviews were followed [21].

Eligibility criteria were designed to be inclusive. Selected settings included time period (all years to 1 January 2020), peer-reviewed English speaking, empirical papers. To explore a wide and occupational therapy-focused range of literature, five databases were selected. These were Web of Science, CINAHL, PsycINFO, OTDBase, and OTseeker. In addition, reference lists and citations of the identified key papers were hand searched. A search strategy was developed by the research team and supported by a specialist librarian. Search terms were as follows: sensory processing OR sensory integration AND parent# OR famil# OR child# AND intervention OR treatment OR therapy OR coaching. Inclusion criteria were parents of children [22] with sensory processing or sensory integration difficulties and parent-focused coaching intervention. Papers were excluded if programs taught the parents an intervention to be applied to the child, e.g., Qi Gong massage [23], as the focus of this review is coaching. Screening and eligibility were undertaken by authors 1 and 4. Level of evidence was identified using Oxford Center for Evidence-Based Medicine Levels of Evidence [24]. Data was charted by two authors using McMaster University's Critical Review Form for Quantitative Studies [25]. This form consists of eight sections (study purpose, literature, design, sample, outcomes, intervention, results, and conclusions or clinical implications), which included questions that prompt the evaluation of the quality of the study.

## 3. Results

**Figure 1:**
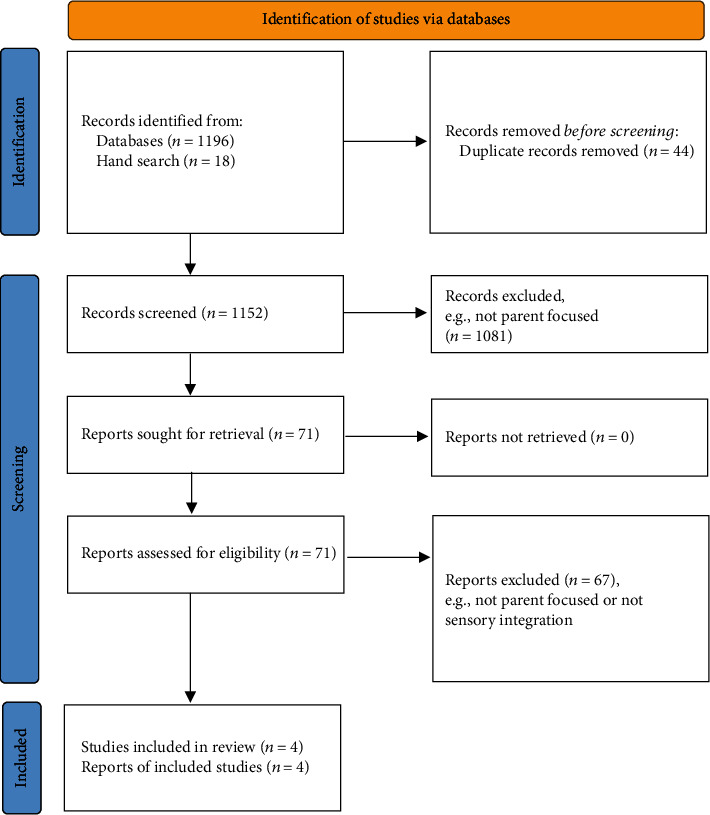
PRISMA 2020 flow diagram. Systematic review of interventions for parents of children with sensory processing and integration difficulties.

As indicated in the Preferred Reporting Items for Systematic Reviews and Meta-Analyses flow diagram (Figure 1) [26], 1196 articles were identified from the databases and a further 18 from hand searches. Following the removal of duplicates, 1152 articles were screened by title and abstract. Seventy-one papers were read in full, to determine relevance to the research questions; of these, four studies met all inclusion criteria, cross-checked by the second author.  Table 1 provides a summary of the final four papers.

### 3.1. What Do These Four Studies Tell Us?

The four studies each used, at least in part, a coaching or problem-solving approach to support parents in developing skills to better manage their children, all of whom had autistic spectrum disorders and sensory integration challenges that caused disruption to daily activities. Three of the four studies were based at home, school, or an environment selected by parents supporting a naturalistic setting. All four studies reported improvements in child behavior or parent knowledge and self-efficacy. Three of the four studies supported parents in their own goal setting. Once the studies started, three studies identified that no dropouts were reported in any of the interventions. Study design was highly variable with evidence between levels 4 and 2 [24]. Participants, when reported, were generally of a higher level of education than the general population. Sample sizes were small, increasing the risk of low generalizability and type II error (failure to reject a false null hypothesis). Follow-up was limited or missing. Demonstration of fidelity to a manualized intervention is present in at least three of the four studies. In the following section, each study is briefly described and appraised in turn; after which, the findings and application to practice are discussed to identify gaps and key areas for future research.

#### 3.1.1. Study 1

Bulkeley et al. [27] aimed to explore the hypothesis that mothers will better manage their child's behavior challenges in the context of daily routines following intervention. Participants were recruited through two child development centers and screened for eligibility for a larger study and then randomly allocated to this intervention. The intervention used a family-centered approach to coaching, based on the principles of sensory integration theory, involving five components adapted from Anzalone and Williamson [28]. These components were observing and reframing sensory processing challenges in context, modifying the environment, modifying the activity, managing the activity, and promoting agency in the child in response to a sensory challenge. Participants were mothers of three children aged three to five, with autism and atypical sensory processing that impacted on behavior and daily routines. Using experimental single case studies with an A-B-A design, the study examined changes in child behavior based on individualized goals selected by the mothers. During the baseline phase (A), mothers rated their perception of the child's target behavior each day using a visual analogue scale (VAS) modified specifically for this study. Following the baseline phase, each mother worked with an occupational therapist for four, hour-long sessions approximately a week apart (B). Mothers completed VAS daily to report their rated perception of the child's targeted behavior. Following the intervention, the baseline phase (A) was repeated without the support of an occupational therapist. Data were graphed and inspected visually, supported by descriptive visual analysis using the median and range of VAS. Improved maternal ratings of child's behavior were observed between the baseline and the end of the intervention phase for all three children; however, improvements were only maintained for one child during the final return to the baseline phase. Strengths of this study include the use of the Infant/Toddler Sensory Profile [29], a standardized tool to identify sensory challenges, supporting the definition of the population being studied. Strategies to support adherence to the intervention protocol or fidelity were described including the use of a checklist against audio recordings of intervention sessions. Finally, the structured use of A-B-A design enabled the isolation of one behavior for measurement demonstrating changes in this small cohort. The primary limitation was the variability in child behavior before the start of the intervention phase.

#### 3.1.2. Study 2

The goal of Dunn et al.'s study [30] was to explore the hypothesis that a contextual occupational therapy intervention delivered individually to families would increase child participation and parent sense of competence to support positive child and family outcomes. Context therapy is an intervention approach that focuses on changing the characteristics of the task and/or environment, rather than the child's impairment, as described by Darrah et al. [31]. The intervention contained three elements: activity settings, daily life routines, and sensory processing patterns. Participants were parents of children with autism, atypical sensory patterns, and self-reported unmet needs in their family life. Working with twenty parents (mother *n* = 19, father *n* = 1), using a pretest/posttest follow-up design, the study examined changes in child behavior (based on individualized goals) and parent competence (based on standardized parent reports of sense of competence and stress). Data were gathered at four time points: four weeks prior to the intervention, at the start of the intervention, at the completion of the ten one-hour sessions of contextual intervention, and, finally, at four weeks' follow-up. Significant improvements in individual self-care, productivity, leisure, and desired behavior goals were reported from pre- to postintervention, which were sustained at follow-up. Post hoc analysis revealed significant differences, with large effect sizes between preintervention and postintervention as well as preintervention to follow-up for individualized child participation goals. In addition, parental defensive (underreporting of difficulties) responding and sense of competence improved significantly from prebaseline to follow-up. Total parental distress demonstrated clinically significant improvement between baseline and follow-up. Strengths included the use of a standardized measure of sensory difficulties, i.e., the Sensory Profile [32], staff training, and a similar manualized procedure reported in a subsequent publication [33].

#### 3.1.3. Study 3

The goal of Gee and Peterson's study [34] was to explore the effectiveness of caregiver psychoeducation groups in increasing the caregiver's perceived and actual knowledge of sensory processing difficulties and their perceived competency in managing the sensory-related behaviors of children with autism. The sample was primarily parents but also included some school staff. The intervention was based on the work of Bailer and Miller [35] and consisted of five sessions of instructional content with one final session focused on a reasoning approach for addressing challenging behaviors related to sensory processing as outlined by Bailer and Miller [35]. No written manual or measurement of fidelity was reported. Outcome measures of parent perceived knowledge, actual knowledge, and self-perceived competence were adapted from previously designed parent survey questionnaires and which demonstrated face validity. A pretest/posttest design was used to measure change between the first and last sessions of intervention. Actual and perceived knowledge of caregivers improved significantly, as did confidence, satisfaction, and feelings of being in control of sensory-related behaviors. However, parents' perception that they possessed the necessary skills to positively manage their child's behavior did not change significantly. Limitations included there being no measure of the child's sensory challenges, limited evidence of the reliability or validity of the study's measurement tools, lack of detail to support replication, and no follow-up.

#### 3.1.4. Study 4

Pashazadeh et al. [36] aimed to identify if Contextual Intervention Adapted for Autism Spectrum Disorder (CI-ASD) promotes child participation in family activities and routines and promotes parenting sense of efficacy. In addition, in Pashazadeh et al.'s study [36], intervention acceptability and participation were explored. The sample was recruited from two rehabilitation centers in Tehran. Parents were randomly allocated to an intervention or wait list control group. Intervention was reported to be based on a wide range of coaching approaches [37–39]. Key characteristics of the intervention are described as (1) sensory processing knowledge, (2) coaching, and (3) social support. No measure of fidelity was reported, but future publication of theoretical underpinnings of the intervention and how the contextual intervention was adapted for this population was reported to be in print. The authors kindly shared the intervention protocol. Data were gathered at three time points: prior to the intervention, at completion of two group and ten one-hour sessions of contextual intervention, and, finally, at four weeks' follow-up. Both the intervention and control groups demonstrated positive changes in individualized participation and functional goals, with statistically significant greater gains in the intervention group between pre- and postintervention and in postintervention to follow-up. Parent self-efficacy was significantly higher in the intervention group versus the control group both at postintervention and follow-up. Attrition was low with 89% of the parents completing the intervention protocol. A treatment acceptability questionnaire demonstrated that most participating parents rated the intervention acceptability as high. Limitations were that the assessment was not blinded and follow-up was short.

## 4. Discussion

This scoping review has identified preliminary but positive findings on the impact of coaching parents of children with sensory integration difficulties. Improved outcomes were observed in child functional skills and behavior as well as parent sense of competence and reduced parent stress. Where measured, the acceptability of one-to-one coaching intervention was reported to be high. However, there are a number of threats to the validity of these findings. Each study took different approaches to the intervention with variety in approach and dosage. It is not therefore possible at this stage to pool data for analysis or to compare the effectiveness of approaches. Only one study used a control group, while the other studies used single case or single cohort designs. Without comparison or further controlled trials, we cannot be confident that the changes did not occur by other mechanisms. Follow-up was either not present or of short duration. Parent and child changes occurred, but we do not have demonstrable evidence that the impact is maintained over more than 4 weeks post intervention.

Transferability of the findings is limited by small underpowered samples, volunteer participants across limited social economic status groups and educational levels. Additionally, all children were reported to have a comorbid diagnosis of autistic spectrum disorder. While a high proportion of children with autistic spectrum disorders experience sensory integration difficulties, not all children with sensory integration difficulties have autistic spectrum disorders [40]. Therefore, we have no evidence as yet that this approach could be applied to a wider cohort of parents of children with sensory integration difficulties that impact participation and function in everyday life.

### 4.1. Points for Practice

Given the preliminary nature of the use of coaching approaches with parents of children with sensory integration difficulties, it is unsurprising that the content of the intervention and ways of working differed. All studies identified the need for shared problem-solving. Information sharing was a component of each of the studies either as a taught component or as a tool for activity analysis and reframing behavior. It is not possible to identify the active ingredients from this review. However, Pashazadeh et al. [36] identified four guiding principles as follows: to situate coaching in everyday life, to seek understanding by working collaboratively with the client, to foster clients' deep thinking about their own life, and to explore resources with clients. Pashazadeh et al. [36] then go on to specify what does and does not constitute Occupational Performance Coaching (OPC). If we consider wider evidence within occupational therapy on Occupational Performance Coaching, the work of Graham et al. [41] provides an alternative detailed description of Occupational Performance Coaching (OPC). They identified three enabling domains of OPC (emotional support, information exchange, and a structured process) and applied techniques of collaborative performance analysis, questioning, listening, observing, modeling, explaining, and in vivo coaching to assist mothers in identifying strategies that support their child's performance. Graham et al. [42] demonstrated preliminary evidence supporting the effectiveness of OPC in improving child and mother occupational performance and mother parenting self-competence in families of children with occupational performance concerns. More recently, Bundy and Bulkeley [43] have expanded the original work of Rush and Sheldon [38] to identify the coaching process as joint planning, action, observation, reflection, and feedback with the addition of reframing (behavior). Robust fidelity to a manualized intervention is key to replicability and evaluating future research.

The goal of occupational therapy is to support participation in daily occupations, and indeed, recent frameworks [17] suggest that the best starting point for an intervention might be goals that focus on participation in a family routine or occupation rather than on behavior. In Bulkeley et al.'s study [27], all child behavioral goals improved, but these improvements were not sustained in two of the three cases following the intervention. While this might stem from the brevity of the intervention with only 4 sessions, the authors suggested that sensory strategies may become embedded more easily in family routines if the goal is focused on increased participation rather than on behavior.

The utility of family participation-based goal setting was demonstrated by Dunn et al. [30] who reported increased participation both at home (e.g., dressing and play) and within the community (e.g., grocery shopping) according to family set goals. Goal setting appears to be an important part of the coaching process. In terms of problem-solving, strategies goal setting helps parents to perceive that they have control and supports change [44, 45]. This empowers parents to be agents of change within their own families [46]. However, goals alone may not be enough, as habits and the environment can act as a barrier to change [47].

There is a need to consider the wider social context or how the social network around the family acts as a barrier or facilitator to potential change. Interestingly, Gee and Peterson [34] involved teachers widening the scope of engagement in intervention for the child and family. There is no evidence from these papers, but other papers consider parent training support engagement with a wider social network [48]. While we can change knowledge and/or intention, this needs to be supported by the empowerment of the individual and their (social) environment in order for that knowledge or intention to be applied to everyday life.

The high attendance rate reported in all studies reviewed is notable (89 to 100%), in comparison with behavioral parent training studies which report attendance ranging from 37% to 98% (mean attendance of 73%) [49]. This may reflect the selective nature of the research samples or a significant level of motivation among parents of children with sensory integration difficulties to engage with intervention opportunities. Pashazadeh et al.'s study [36] explored engagement by identifying the acceptability of the intervention to participants. High acceptability further supports the value of this coaching approach to parents.

The papers reviewed considered both group and individual approaches. It is difficult to compare the impact of group versus individual intervention in this context due to the lack of detail of content and variety in dosage of interventions. While individual intervention allows focus on family-centered goals, there are advantages of a group intervention in terms of access to peers and social support networks. Group parent intervention may be more readily available in resource-restricted contexts. Wymbs et al. [50] surveyed parents of children with ADHD and found that 85% choose group intervention over paper information. A further study that considered group and follow-up individual parent intervention for parents of children with autistic spectrum conditions supported the social validity of the group-based approach [51]. However, the message from both papers was that the group intervention did not substitute the need for direct one-to-one intervention for the parent or child.

Three studies explored intervention in home, school, or parent-selected setting away from home and one study was based in a clinic environment. The move towards intervention in a more naturalistic environment reduces the demands for time, travel, and finance on families who are already experiencing additional demands and is advocated in the wider occupational therapy literature [31]. Interventions varied in length from four to ten hours for individual coaching and six hours for group parent/teacher training. Bulkeley et al. [27] suggest that a longer period of intervention may be required to sustain changes postintervention than the 4 hours of intervention in their study. Indeed, Vismara et al. [52] found that parents acquired strategies by the fifth to sixth hour of a 12-hour group-based education program for parents of autistic children. This would suggest that four hours of intervention may be too little to support sustained changes, while six hours plus may be more effective in facilitating longer term strategies, but this assertion has not been tested with parents of children with sensory integration difficulties.

### 4.2. Limitations

From the perspective of the studies reviewed, there are a number of limitations. Most of the studies rely on volunteers which may limit the generalizability of findings. Sample size for the combined studies remains small. Data collection was either parent led or therapist led and nonblinded. Findings are limited by the lack of control in three of the four studies. Methods of data collection varied, although three of the four studies considered goals that were individualized to the child or family. From the perspective of the scoping review, there are also a number of limitations. Due to the variety of approaches to intervention, it is not possible at this point to compare or combine data from these articles. As a scoping review, this paper provides an initial mapping of the information currently available.

### 4.3. Gaps in the Literature and Areas for Further Research

All studies reviewed identified the need for further research in this area. Three of the four studies incorporated an element of parent goal setting enabling parents to identify and address parents' own agenda. However, parents were not invited to indicate their own preferences in terms of support delivery. Identification of what support parents do or do not value provides the foundation for meeting parent-identified needs. Do parents want support, and if so, what is their preferred format to receive it?

Design of interventions was based on coaching literature and sensory integration knowledge and understanding. It is difficult to identify from the current studies which aspect promotes change in child or parent outcomes. In order to identify the contribution of each component of intervention, it may be pertinent to consider the impact of sharing information on sensory integration versus only coaching parents without framing within the context of sensory integration concerns. A manual of the intervention is important to demonstrate replicability. Evaluation of consistency between manual of intervention and actual intervention scored by an independent assessor can assure implementation fidelity. Evidence of group parent intervention is scant with only informal measurement of parent knowledge and competence. There is a need to consider the impact of group versus individual intervention. Given the differences in staff costs and the additional benefits of peer support, further investigation of group-based parent coaching might be considered as an area for further exploration.

In measuring outcomes, there is inconsistency. Group-based intervention may have an impact on parent stress and coping alongside child functioning and the wider health outcome of family participation, but this has not yet been explored.

Parent coaching needs to be considered in the context of the range of services available to families [16, 17]. Reynolds et al. [16] see coaching as one piece of multifaceted intervention. This assertion is supported by the findings of Rivard et al. [51] who suggest that parent coaching while children were on a waiting list provide benefits to the child, but may be detrimental to parent stress if child-focused early intervention services are not concurrently available [51]. In contrast, Ashburner et al. [17] suggest coaching as an appropriate starting point alongside universal design approaches. In addition to the evidence base, health economic drivers and context of delivery will influence approaches to service delivery. That is, coaching provides one approach to intervention that may also include, for example, direct intervention to the child or adaption to the environment. At present, we do not have sufficient evidence to comment on whether concurrently using multiple interventions, sequencing a series of interventions, or utilizing a single intervention in isolation best meets the needs of children and their families as no direct comparison studies have been undertaken. At this stage, it is not about one approach to intervention replacing another but understanding what each approach contributes.

Finally, there is little long-term follow-up and application to wider social economic status participants would increase our understanding of the relative merits of a parent coaching intervention.

## 5. Conclusions

These studies demonstrate that it is possible in families of children with sensory integration difficulties to increase a parent's knowledge and understanding through education but that approaches benefit from a coaching perspective so that the knowledge gained can be applied to everyday life. It is also possible to improve child behavior, reduce parental stress, and increase a parent's sense of competence through parental coaching. Evidence for one-to-one intervention with parents of children with SID is limited but promising. There is scant evidence to support group parent intervention in parents of children with sensory integration difficulties.

## Figures and Tables

**Table 1:** Summary of papers.

Authors	Design	LoE^∗^ (20)	Sample	Format and environment	Frequency, intensity, and duration	Approach	Outcome measures	Results
Bulkeley et al. [27]	Single case experiment design using an ABA protocol	4	*n* = 3 mothers of children with autism; age range 3-5 y Child sensory issues identified through the Infant/Toddler Sensory Profile	Individual family-centered coaching based on principles of sensory integration theory as their initial intervention Environment: home	4 × 1 hour per intervention, 1 week or more apart	Brief family-centered coaching using a sensory-based framework	Visual analogue scale for target individual behaviors (1) Tolerance of the sound of a hairdryer (2) Reduced rigid eating behavior (3) Reduced reckless and dangerous behavior	All three participants showed positive response to sensory-based intervention. Maintenance of gains was evident in only one case.
Dunn et al. [30]	Repeated measure pretest/posttest (1) Baseline (2) Preintervention (3) Postintervention (4) 4-week follow-up	3	Individual family intervention; *n* = 20 (19 mothers, and one father) of children with autism spectrum disorders; age range 3-10 y Child sensory issues reported through Sensory Profile [32]	Individual family-centered coaching The intervention reflected principles of context therapy Environment: a location of their choosing, in person or via telehealth	10 × 1-hour weekly sessions with some flexibility to meet scheduling needs	Coaching in family context within daily routines informed by the child's sensory patterns	(1) Individual goals: Canadian Occupational Performance Measure (COPM); Goal Attainment Scaling (GAS); e.g., dressing, eating, having injections, riding in car, and transitioning for bus to home (2) Parent Stress Index-Short Form (PSI-SF) (3) Parent Sense of Competence Scale (PSOC)	(1) Individualized COPM and GAS goals demonstrated a significant improvement between pretest and posttest, sustained at 4-week follow-up (2) PSI-SF: significant improvement in parental defensiveness and parental distress from measurement 1 to 4 (3) Significant increase in parent sense of efficacy from first to last assessment?
Gee and Peterson [34]	Pretest-posttest (1) Week 1 (2) Week 6	3	*n* = 7 parents, *n* = 3 teachers of children on the autism spectrum; age range 5 to 10 years Child sensory issues reported by parent or through child Short Sensory Profile [53]	Group intervention Environment: conference room in a college campus	6 × 1 hour per week 6 weeks	Education Weeks 1–5: promoting parent's knowledge Week 6: promoting problem-solving (A SECRET [54])	Adapted parent completed questionnaires on (1) Self-perceived knowledge of sensory processing concepts (2) Actual knowledge of sensory processing concepts (3) Self-rated competency for dealing with children exhibiting behaviors related to sensory processing disturbances and/or disorders	(1) Significant increase in self-perceived knowledge (2) Significant increase in actual knowledge (3) Significant increase in confidence and satisfaction in own ability to handle child's behavior; no significant change in belief that ability to deal with sensory-related behaviors had a positive impact
Pashazadeh et al. [36]	Randomized control trial	2	Parents of children diagnosed with autistic spectrum disorder with at least one sensory pattern outside the typical range as measured by Short Sensory Profile II [55]; *n* = 15 intervention group, *n* = 16 wait list control group	Group sessions plus individual coaching sessions Environment: rehabilitation center	2 sessions of group training sessions plus 10 × 45-minute weekly individual session	Three treatment characteristics (1) Sensory processing knowledge (2) Coaching (3) Social support	(1) COPM (2) GAS (3) Parent sense of efficacy measure	(1) Significant increases in child participation, performance/satisfaction, and individualized goal attainment for the intervention group as compared to the control group, sustained at 4 weeks of follow-up (2) Significant increase in the intervention group of parent self-efficacy from pre- to postintervention, maintained at 4 weeks

LoE^∗^: levels of evidence.

## Data Availability

The review data used to support the findings of this study are available from the corresponding author upon request.
